# Outcome at hospital discharge of patients admitted to the icu. analysis of a prospective cohorts from a polyvalent icu in an university hospital

**DOI:** 10.1186/2197-425X-3-S1-A964

**Published:** 2015-10-01

**Authors:** D Arias-Verdú, M Delgado-Amaya, G Seller-Pérez, E Banderas-Bravo, J Barrueco-Francioni, M Herrera-Gutierrez

**Affiliations:** Complejo Universitario Carlos Haya, Malaga, Spain

## Objectives

To define the outcome at hospital discharge of our patients and detect possible variables differentiating mortality intra-ICU and after ICI-discharge.

## Methods

Preliminary results from a prospective cohorts study over all patients admitted to our unit for one year excluding coronary patients and those with a stay of less that 24 hours. The Ethics Committee from our centre approved this work. Data as mean (standard error for mean). We applied a logistic regression methodology. Results as OR (95% CI).

## Results

962 patients. Admitted from emergencies dept. 28%, hospital wards 23,9%, surgical theatre 38,2% (9,3% urgent y 28,9% elective) y 10% from other centres. 38,1% had been admitted to the hospital at least once the previous year and 5,6% in the ICU. Hospital stay previous to ICU admission was 5,9(0,36) days [5,26 (0,32) in survivors, 8,42(1,13) in deaths, p < 0,001].

Total mortality was 23%, 18,1% during UCI stay and the rest after discharge (3,7% in ICU readmission and 1,1% in ward). Among 41 cases with a DNR order, 31,7% was discharged alive from the hospital.

Age (OR 1,01, 1-1,02), previous cerebral vascular disease (3,8, q,8-8,1, immunosuppression (1,8, 1,2-2,8) and SOFA at admission (1,3, 1,2-1,4), mechanical ventilation (5,2, 2,7-9,7), vasopressors (1,8, 1,2-2,7), SOFA > 6 (2,3, 1,4-3,3) or AKI (3,4, 2,3-5,4) were related to mortality.

When focusing in mortality after ICU discharge, male gender (OR 2,6, IC 1,1-6,2), admission from a medical ward (OR 9,0, IC 3,2-25,6), hospital stay before admission (OR 1,03, IC 1,01-1,05), AKI (OR 3,1, IC 1,1-8,2) and Sabadell score (Grade I, OR 10,6:3,3-33,9; II 27,4:7,8-96,5; III 130,9:32,1-534,2) showed relationship.

## Conclusions

Among our patients DNR order status was registered in a low number of cases and a third of those with a DNR order were discharged alive from the hospital.

The Sabadell Score is a useful tool for predicting hospital mortality in those patients discharged from the ICU.

